# Supplementary material for: Genotypic and phenotypic prevalence of Nocardia species in Iran: First systematic review and meta-analysis of data accumulated over years 1992-2021
Source: PLoS One. 2021 Jul 22;16(7):e0254840. doi: 10.1371/journal.pone.0254840 (PMC8297923; doi:10.1371/journal.pone.0254840)
Supplement: S1 Checklist — (DOC) [file pone.0254840.s001.doc]

| **Section/Topic** | **#** | **Checklist Item** | **Reported on Page #** |
| --- | --- | --- | --- |
| **TITLE** | | | |
| Title | 1 | **Genotypic and Phenotypic prevalence of Nocardia species in Iran: First systematic Review and Meta-Analysis of data accumulated over years 1992-2020** | 1 |
| **ABSTRACT** | | | |
| Structured summary | 2 | Background  Nocardia species belong to the aerobic actinomycetes group of bacteria which are gram-positive and partially acid fast Bacilli. These bacteria may sometimes be associated with nosocomial infections. Nocardia diseases are not required to be reported to public health authorities in Iran. Hence, the present study was designed to determine the prevalence of human Nocardia spp. in Iran by using a systematic review and meta-analysis according to the preferred reporting items for systematic reviews and meta-Analyses statement.  Methods  The data of the prevalence of Nocardia species were collected from databases such as Embase, PubMed/MEDLINE via Ovid, Web of Science, Scopus and Google Scholar as well as national Iranian databases, including SID, Magiran. Analyses were conducted by STATA 14.0.  Results  The meta-analyses showed that the proportion of Nocardia spp. in Iranian studies varied from 1.71(1.17, 2.24) to 0.46(0.09, 0.83). N. asteroides (21% [95% CI 1.17, 2.24]), N. cyriacigeorgica (17% [95% CI 0.99, 1.77]), N. facanica (10% [95% CI 0.75, 1.00]) were considered to be common causative agents  Conclusions  Our study presents that Nocardia spp. are re-emerging on account of an increase in the number of Iranian populations. Considering our results, the establishment of advanced diagnostic facilities for the rapid detection of Nocardia infections are required for optimal therapeutic strategies of Nocardia spp. in Iran. Our findings could help the programmatic management of the disease within the context of Nocardia control programmers.  Systematic Review number: PONE-D-21-07360R1 | 3 |
| **INTRODUCTION** | | | |
| Rationale | 3 | *Nocardia* species (spp.) belong to the aerobic actinomycetes group of bacteria which are gram-positive and partially acid‑fast bacilli (AFB) [1]. These bacteria are saprophytic and are found in soil and water, however, they may sometimes be associated with nosocomial infections [2]. More than 40 of the approximately 86 *Nocardia* spp. characterized, have been involved in human infections and include *Nocardia asteroides complex* (more than 50% human cases), *N. brasiliensis, N. abscessus, N. cyriacigeorgica, N. farcinica, N. nova, N. transvalensis complex, N.* *novacomplex, N. pseudobrasiliensis*, and the recently described spp. include *N. veterana , N*. *paucivorans , N. elegans , N. wallacei* and *N. blacklockiae* of the *N. transvalensis complex* [3].   Human *Nocardia* infection can be airborne via inhalation of dust particles (pulmonary nocardiosis-pneumonia, lung abscess) or skin infections (cutaneous nocardiosis-cellulitis, ulcers), and the infection can then disseminate to the brain, kidneys, joints, heart, eyes, and bones [4]. So far, person to person transmission is not documented [3].  Pulmonary nocardiosis is a low but severe opportunistic infectious disease and mainly affects patients with compromised cell-mediated immunity, such as those experiencing long-term steroid use, patients with acquired immunodeficiency syndrome (AIDS), or recipients of organ transplantation [5]. However, early diagnosis of pulmonary nocardiosis may be difficult because signs and symptoms in the affected patients are nonspecific and very similar to tuberculosis (TB) [6].  Co-infection with *Nocardia* spp. in patients suffering from mycobacterial lung infection, highlights the importance of laboratory diagnosis that may facilitate better patient management. The diagnosis of nocardiosis is currently based on direct examination and conventional culture, while serology is usually not useful [7]. The molecular methodologies which have provided precise identification of *Nocardia* spp. are important for studies of clinical and epidemiological investigations [8]. Unlike tuberculosis, *Nocardia* diseases are not required to be reported to public health authorities in Iran, and therefore, precise incidence and prevalence data are not available. In order to estimate what the true burden of *Nocardia* human infections is global, a meta-analysis was performed to review all studies related to the epidemiology of the *Nocardia* causative agents. | 4-6 |
| Objectives | 4 | The present study was designed to determine the prevalence of human *Nocardia* spp. In iran by using a systematic review and meta-analysis according to the preferred reporting items for systematic reviews and meta-analyses statement. | 5-6 |
| **METHODS** | | | |
| Protocol and registration | 5 | file:///C:/Users/pc/Downloads/JBI_Critical_Appraisal-Checklist_for_Analytical_Cross_Sectional_Studies2017_0.pdf | 6 |
| Eligibility criteria | 6 | After the search results were merged into Endnote (X7; Thomson Reuters), the resultant was de-duplicated and screened by applying an Rayyan Qatar Computing Research Institute online application. Criteria considered for inclusion were cross-sectional surveys assessing the frequency or prevalence of *Nocardia* spp. in Iran. Based on the research protocol and the eligibility criteria, the titles and abstracts were separately retrieved by two independent researchers. Following the elimination of repetitive studies, the full text of the papers in terms of eligibility criteria and the required extracted necessary information were studied. Disagreements between the two researchers were resolved by a consensus method. The final data extracted from the search results included corresponding author, year, place, research design, sample size, location, study period, individual *Nocardia* spp., and detection method. The exclusion criteria were including the papers with the following features: review articles, meta-analyses or systematic reviews, case reports and letter to editor studies, congress abstracts, and the duplication papers, as well as articles in languages other than English or Persian and those available only in abstract form. To evaluate the eligibility of the articles with inadequate information, we made a contact with the corresponding author. Culture as well as biochemical and molecular testes were the standard methods for detection. | 6-7 |
| Information sources | 7 | A systematic review of available literature was searched using the electronic database such as: Embase, PubMed/MEDLINE via Ovid, Web of Science, Scopus and Google Scholar as well as national Iranian databases, including SID, Magiran, with medical subject headings (MeSH) terms and a proper use of keywords. The search strategy was as follows: " *Nocardia* ", "nocardiosis "," *Nocardia* and human infection", "actinomycete" and "Iran". Original articles on *nocardia* and a time filter (from August 1992 to January 2021) applied including Persian and English articles were considered. Likewise, the full texts of potentially relevant articles were assessed for eligibility independently and in duplicate by two investigators. In addition to articles published in English, we also looked for relevant articles in Persian. | 6-7 |
| Search | 8 | Search key words: " *Nocardia* ", "nocardiosis "," *Nocardia* and human infection", "actinomycete" and "Iran". | 6-7 |
| Study selection | 9 | A total of 93 articles were obtained by a literature search with a combination of keywords from the databases as shown in. In secondary screening and after duplication, 18 articles were identified and were removed due to the irrelevant titles. Then based on the abstract evaluation, 55 articles were excluded (3 review articles, 27 case reports, 8 letters to the editor, and 17 were related to non-clinical *Nocardia* specimens). So, according to quality assessment criteria and inclusion/exclusion criteria data, a remaining 20 most-related articles were included in present study Among 20 articles involving a total of 338 *Nocardia* isolates, the prevalence of *Nocardia* spp. were recorded. The articles were published between years 2000 to 2020. | 6-7 |
| Data collection process | 10 | Two reviewers independently extracted the data from eligible studies. According to inclusion and exclusion criteria, all collected data from the selected studies were tabulated as follows: (1) First author, (2) publication date, (3) enrollment time, (4) province of study, (5) all patients included in study, and (6) prevalence of *Nocardia* human infections. Two authors extracted data from involved studies independently. Inconsistency between the reviewers was resolved through discussion. | 7 |
| Data items | 11 | The quality of papers was evaluated using the Strengthening the Reporting of Observational studies in epidemiology (PRISMA) checklist and the guidelines of the Cochrane Handbook for Systematic Reviews and Interventions [9]. This checklist has 8 parts which covers different sections of reports. If necessary, the authors were contacted for further information. | 6 |
| Risk of bias in individual studies | 12 | In this study, the prevalence of *Nocardia* in the country was collected and then the variance of each study was determined by Double arcsine conversion method. The point estimates of effect size, the prevalence of *Nocardia* spp., and its 95% confidence interval (95% CI) were estimated for each study. Random effects models were used, taking into account the possibility of heterogeneity between studies, which was tested with the Cochran’s Q- and the I2 statistics. In order to assess possible publication bias, Egger weighted regression methods were used. Value of P < 0.05 was considered indicative of statistically significant publication bias. Analyses were conducted by STATA 14.0 (StataCorp, College Station, TX, US) | 8 |
| Summary measures | 13 | In total, 42 different *Nocardia* spp. were identified in the studied Iranian articles, with the varied proportion from 1.71(1.17, 2.24) to 0.46 (0.09, 0.83) using 90% confidence interval. *N. asteroides* (21% [1.17, 2.24]), *N. cyriacigeorgica* (17% [95% CI 0.99, 1.77]), *N. facanica* (10% [0.75, 1.00]) were considered to be the most common causative agents, while, *N. coubleae* (0/0011% [0.09, 0.80]), *N. cummidelens* (0/0011% [0.09, 0.80]), *N. neocaledoniensis* (0/0011% [0.24, 1.84]) and *N. ignorata* (0/0011% [0.09, 0.80]) isolates were considered as the uncommon causative agents mentioned in only one study. It is necessary to mention that the causative agents were not identified to the spp. level in 12% of cases [01.67, 2.86]). | 8 |
| Synthesis of results | 14 | In total, 42 different *Nocardia* spp. were identified in the studied Iranian articles, with the varied proportion from 1.71(1.17, 2.24) to 0.46 (0.09, 0.83) using 90% confidence interval. *N. asteroides* (21% [1.17, 2.24]), *N. cyriacigeorgica* (17% [95% CI 0.99, 1.77]), *N. facanica* (10% [0.75, 1.00]) were considered to be the most common causative agents, while, *N. coubleae* (0/0011% [0.09, 0.80]), *N. cummidelens* (0/0011% [0.09, 0.80]), *N. neocaledoniensis* (0/0011% [0.24, 1.84]) and *N. ignorata* (0/0011% [0.09, 0.80]) isolates were considered as the uncommon causative agents mentioned in only one study. It is necessary to mention that the causative agents were not identified to the spp. level in 12% of cases [01.67, 2.86]). | 8 |

Page 1 of 2

| **Section/Topic** | **#** | **Checklist Item** | **Reported on Page #** |
| --- | --- | --- | --- |
| Risk of bias across studies | 15 | No bias was observed in the studies | 8 |
| Additional analyses | 16 | **Statistical analysis**  In this study, the prevalence of *Nocardia* in the country was collected and then the variance of each study was determined by Double arcsine conversion method. The point estimates of effect size, the prevalence of *Nocardia* spp., and its 95% confidence interval (95% CI) were estimated for each study. Random effects models were used, taking into account the possibility of heterogeneity between studies, which was tested with the Cochran’s Q- and the I2 statistics. In order to assess possible publication bias, Egger weighted regression methods were used. Value of P < 0.05 was considered indicative of statistically significant publication bias. Analyses were conducted by STATA 14.0 (StataCorp, College Station, TX, US) | 8 |
| **RESULTS** | | | |
| Study selection | 17 | A total of 93 articles were obtained by a literature search with a combination of keywords from the databases as shown in Fig. 1. (**Fig 1. Flow diagram of literature search, within the manuscript text)** | 8-9 |
| Study characteristics | 18 | In secondary screening and after duplication, 18 articles were identified and were removed due to the irrelevant titles. Then based on the abstract evaluation, 55 articles were excluded (3 review articles, 27 case reports, 8 letters to the editor, and 17 were related to non-clinical *Nocardia* specimens). So, according to quality assessment criteria and inclusion/exclusion criteria data, a remaining 20 most-related articles were included in present study [10-29]. Among 20 articles involving a total of 338 *Nocardia* isolates, the prevalence of *Nocardia* spp. were recorded. The articles were published between years 1994 to 2021. | 9,11 |
| Risk of bias within studies | 19 | In order to assess possible publication bias, Egger weighted regression methods were used. Value of P < 0.05 was considered indicative of statistically significant publication bias. Analyses were conducted by STATA 14.0 (StataCorp, College Station, TX, US) | 9,12,15 |
| Results of individual studies | 20 | In total, 338 different *Nocardia* spp. were identified in the studied Iranian articles, with the varied proportion from 1.71(1.17, 2.24) to 0.46 (0.09, 0.83) using 90% confidence interval Table 2. *N. asteroides* (21% [1.17, 2.24]), *N. cyriacigeorgica* (17% [95% CI 0.99, 1.77]), *N. facanica* (10% [0.75, 1.00]) were considered to be the most common causative agents, while, *N. coubleae* (0/0011% [0.09, 0.80]), *N. cummidelens* (0/0011% [0.09, 0.80]), *N. neocaledoniensis* (0/0011% [0.24, 1.84]) and *N. ignorata* (0/0011% [0.09, 0.80]) isolates were considered as the uncommon causative agents mentioned in only one study. It is necessary to mention that the causative agents were not identified to the spp. level in 12% of cases [01.67, 2.86]). Fig. 2 shows the forest plot of meta-analysis of *Nocardia* prevalence. Some evidence for publication bias was observed in Fig. 3. | 12-15 |
| Synthesis of results | 21 | Out of these 20 articles, 15 were belong to reports from Tehran, center of Iran. The rest were as follows: 5 from southwest of Iran (4 of them from Khuzestan and 1 from Kermanshah provinces), Isfahan, Yazd, Central (Arak), and Golestan provinces one report each. Fig. 4 shows the distribution of *Nocardia* spp. in different parts of Iran. The *Nocardia* isolation in the central provinces of Iran demonstrated apparent characterization, as from the central province (Tehran) to the southwest province (Khuzestan, Kermanshah) the *Nocardia* isolation rate was increased. The most frequent isolated *Nocardia* spp*.*  in central provinces were *N. otitidiscaviarum.caviae*and *N. cyriacigeorgica,*while *N. farcinica* and *N. wallacei* were the most typical isolated species (Table 3). | 12-15 |
| Risk of bias across studies | 22 | Please refer to 19. | 15 |
| Additional analysis | 23 | The distribution of pulmonary nocardiasis sites shown in Table 4. Among the patients with pulmonary Nocardia infection 139 out of 259 (53%) with bronchoalveolar lavage (BAL) and 120(46%) was sputum. Among the patients with extra pulmonary specimen, 4(2%) with wound, 26 (32%) absecus, 8 (27%) blood, one specimen was pleural and 30 (26%) skin. The distribution of extra pulmonary sites shown in Table 5. | 15 |
| **DISCUSSION** | | | |
| Summary of evidence | 24 | The analysis has revealed a relatively high prevalence of *Nocardia* spp. among Iranian patients. The overall prevalence of this species was also greater when the study was performed after the year 2000 with the rate of 54% compared to the rate before year 2000 as 38%. The higher number of reports of *Nocardia* spp. appears to be due to the significance of the disease identified by microbiologists and physicians, advancement of laboratory facilities, and inceasing the incidence of immunocompromised hosts, which gives rise to a growth in *Nocardia* spp. in the entire population. In general, the relatively high incidence (0.49%) of *Nocardia* spp. in our country may have an adverse impact on public health. Despite the implementing of national control programs, tuberculosis (TB) is still among the highest health hazard in Iran. Owing to the clinical similarity of Nocardiosis to many other infections, TB in particular, *Nocardia* infections are commonly missed/ or not suspected and delay in diagnosis [33]. In our study, *N. asteroides* was the most frequent isolate species, which supports the findings of other research works. | 16-17 |
| Limitations | 25 | A vast majority of surveys have been performed in the central and southwest regions, but not northern part, of Iran. These data denote that Tehran, the capital city of Iran with many healthcare centers, has a referral role for the whole areas of the country. Therefore, patients, especially those with complicated conditions, are referred to Tehran from all over the country for better management. Considering these data, it seems that the main reason for the isolation of most *Nocardia* spp. in the central provinces is accessibility of the commercial methodologies for detecting this species. Thus, conducting continuous DNA sequencing of homologous genes with a maximum resolution is considerably recommended for areas having a high incidence of *Nocardia* spp. [40]. | 18 |
| Conclusions | 26 | Taken together, our study presents that Nocardia spp. are re-emerging for the sake of an increased number of Iranian populations. Our findings could help the programmatic management of the disease within the context of Nocardia control programmers. This study emphasizes that the survey of Nocardia spp. and infections related to this bacterium has been neglected and should be studied in the future. Additionally, the need to express patients' clinical information in studies can assist in providing better results in future investigations. The distinction of Nocardia infection from other pulmonary infections such as TB has significant practical importance. Furthermore, when the infection is clinically considerable, the selection of optimal treatment regimens should be bore in mind by physicians. Considering our results, the establishment of advanced diagnostic facilities for the rapid detection of Nocardia spp. are necessary for optimal therapeutic strategies of Nocardia infections in Iran. | 18 |
| **FUNDING** | | | |
| Funding | 27 | This work is part of a research project which was approved in Infectious and Tropical Diseases Research Center, Ahvaz Jundishapur University of Medical Sciences, Ahvaz, Iran, and was supported by a grant ( No.: OG-9829 ) from research affairs of the university. | 19 |

*From:* Moher D, Liberati A, Tetzlaff J, Altman DG, The PRISMA Group (2009). Preferred Reporting Items for Systematic Reviews and Meta-Analyses: The PRISMA Statement. PLoS Med 6(6): e1000097. doi:10.1371/journal.pmed1000097

For more information, visit: **www.prisma-statement.org**.

Page 2 of 2
