# Supplementary material for: Genotypic and phenotypic prevalence of Nocardia species in Iran: First systematic review and meta-analysis of data accumulated over years 1992-2021
Source: PLoS One. 2021 Jul 22;16(7):e0254840. doi: 10.1371/journal.pone.0254840 (PMC8297923; doi:10.1371/journal.pone.0254840)
Supplement: S1 Data — (DOCX) [file pone.0254840.s002.docx]

We hereby confirm that our submission contains our "minimal data set", which PLOS defines as consisting of the data set used to reach the conclusions drawn in the manuscript with related metadata and methods, and any additional data required to replicate the reported study findings in their entirety. This includes:
- The values behind the means, standard deviations and other measures reported;

- The values used to build graphs;
- The points extracted from images for analysis.

**Results of Nocardia Prevalence Meta-Analysis**

In this study, the prevalence of Nocardia in the country was collected and then the variance of each study was determined by the double arcsin conversion method. Point estimation The effect size of Nocardia prevalence and 95% confidence interval (95% CI) were estimated for each study. Cochran Q test and I2 index were used to evaluate the heterogeneity of the studies. Due to the heterogeneity between the studies, random effects models were used to combine the studies. Egger regression model was used to determine the presence of diffusion bias. In order to perform meta-analysis, software (STATA ver14) was used. Significance level was considered P <0.05 in the tests

**Table 6. Meta-analysis of the prevalence of *Nocardia* in Iran**

| Egger test  P-Value | Egger test  t | Heterogeneity test ،P-Value | Heterogeneity test ،I2(%) | Prevalence of nocardia  (95% CI) | N. of studies | studies |
| --- | --- | --- | --- | --- | --- | --- |
| 0.865 | 0.17 | <0.001 | 93.7 | 0.49(0.37, 0.60) | 20 | All studies |
| 0.493 | -0.75 | 0.154 | 37.8 | 0.38(0.30, 0.45) | 6 | Studies before 2010 |

Using the results obtained from Table (1), the heterogeneity test shows that there is heterogeneity between studies (I2 = 93.7%, P <0.001). Therefore, a random effects model was used to estimate the prevalence of *Nocardia.* Egger test shows that there was no bias in the dissemination of results (t = 0.17, P = 0.865). The estimated overall prevalence of Nocardia in Iran was 0.49% with a 95% confidence interval (0.37, 0.60).

**Fig. 2.**

**Forest plot of meta-analysis of *Nocardia* prevalence in Iran based on random‑effect models.**

The diagram above (Fig. 2), shows the forest plot meta-analysis of *Nocardia* prevalence in various studies in Iran. This diagram shows the information of each study and their final results. The estimate of each study is represented by a square and its 95% confidence interval is represented by a transverse line. Also, the overall outcome of the prevalence of *Nocardia* in Iran is rhombic at the bottom of the accumulation graph (Fig. 3.).

**Fig. 3.**

**Funnel plot of the meta-analysis on prevalence of *Nocardia* species**

**Table 2. *Nocardia* spp. distribution among Iranian studies**

| Nocardia spp. | N. of studies | N/% | Prevalence of *nocardia*  (95% CI*) | Heterogeneity test I^2^ (%) | Heterogeneity test *P*-Value | Eggers test t | Eggers test *p-* value |
| --- | --- | --- | --- | --- | --- | --- | --- |
| ***N. asteroides*** | 9 | 74/(21%) | 1.71(1.17, 2.24) | 92.8 | <0.001 | 1.34 | 0.228 |
| ***Nocardia.spp*** | 8 | 42/(12%) | 2.27(1.67, 2.86) | 78.7 | <0.001 | 0.78 | 0.470 |
| ***N. cyriacigeorgica*** | 6 | 60/(17%) | 1.38(0.99, 1.77) | 86.0 | <0.001 | 1.84 | 0.139 |
| ***N. farcinica*** | 6 | 41/(12%) | 0.87(0.75, 1.00) | 0.0 | 0.562 | -0.06 | 0.956 |
| ***N. otitidiscaviarum.caviae*** | 5 | 40/(11%) | 0.66(0.49, 0.82) | 30.0 | 0.222 | 0.58 | 0.601 |
| ***N. nova*** | 5 | 7/(0/02) | 0.38(0.25, 0.50) | 25.7 | 0.250 | 7.11 | 0.006 |
| ***N. wallacei*** | 3 | 14/(0/04) | 0.70(0.35, 1.05) | 71.2 | 0.031 | 3.12 | 0.198 |
| ***N. arthritidis*** | 3 | 3/(0/008) | 0.28(0.14, 0.42) | 0.0 | 0.442 | 1.86 | 0.145 |
| ***N. a.complex*** | 2 | 5/(0/014) | 1.22(0.80, 1.64) | 65.0 | 0.091 | - | - |
| ***N. carnea*** | 2 | 6/(0/017) | 0.39(0.24, 0.54) | 39.9 | 0.197 | - | - |
| ***N. kruczakiae*** | 2 | 2/(0/008) | 0.25(0.10, 0.40) | 0.0 | 0.411 | - | - |
| ***N. abscessus*** | 2 | 33/(0/09) | 0.59(0.16, 1.03) | 85.5 | 0.009 | - | - |
| ***N. veterana*** | 2 | 2/(0/008) | 0.25(0.10, 0.40) | 0.0 | 0.411 | - | - |
| ***N. brasiliensis*** | 2 | 2/(0/008) | 0.53(0.22, 0.84) | 0.0 | 0.351 |  |  |
| ***N. transvalensis*** | 1 | 1/(0/0011) | 0.46(0.09, 0.83) | - | - | - | - |
| ***N. coubleae*** | 1 | 1/(0/0011) | 0.44(0.09, 0.80) | - | - | - | - |
| ***N. cummidelens*** | 1 | 1/(0/0011) | 0.44(0.09, 0.80) | - | - | - | - |
| ***N. ignorata*** | 1 | 1/(0/0011) | 0.44(0.09, 0.80) | - | - | - | - |
| ***N. mexicana*** | 1 | 2/(0/008) | 1.40(0.60, 2.20) | - | - | - | - |
| ***N. neocaledoniensis*** | 1 | 1/(0/0011) | 1.04(0.24, 1.84) | - | - | - | - |

*Confidence Internal

**Fig 4. Forest chart of meta-analysis of *Nocardia* prevalence by provinces of Iran based on random effects model.**
