# Supplementary material for: Genotypic and phenotypic prevalence of Nocardia species in Iran: First systematic review and meta-analysis of data accumulated over years 1992-2021
Source: PLoS One. 2021 Jul 22;16(7):e0254840. doi: 10.1371/journal.pone.0254840 (PMC8297923; doi:10.1371/journal.pone.0254840)
Supplement: Attachment — Submitted filename: Response to Reviewers.docx [file pone.0254840.s004.docx]

**Response to Reviewers’ comments**

**PONE-D-21-07360
Reviews article
Genotypic and Phenotypic diversity *Nocardia* species in Iran: First systematic Review and Meta-Analysis of data accumulated over years (1992-2020)
PLOS ONE**

***Note: all corrections are Red-written in this file and Revised Manuscript file with Track Changes.**

A major revision is needed.


Please submit your revised manuscript by Jun 27 2021 11:59PM. If you will need more time than this to complete your revisions, please reply to this message or contact the journal office at plosone@plos.org. When you're ready to submit your revision, log on to https://www.editorialmanager.com/pone/ and select the 'Submissions Needing Revision' folder to locate your manuscript file.

Please include the following items when submitting your revised manuscript:

- A rebuttal letter that responds to each point raised by the academic editor and reviewer(s). You should upload this letter as a separate file labeled 'Response to Reviewers'.
- -is done
- A marked-up copy of your manuscript that highlights changes made to the original version. You should upload this as a separate file labeled 'Revised Manuscript with Track Changes'.
- -is done
- An unmarked version of your revised paper without tracked changes. You should upload this as a separate file labeled 'Manuscript'.
- -is done

If you would like to make changes to your financial disclosure, please include your updated statement in your cover letter. Guidelines for resubmitting your figure files are available below the reviewer comments at the end of this letter.

If applicable, we recommend that you deposit your laboratory protocols in protocols.io to enhance the reproducibility of your results. Protocols.io assigns your protocol its own identifier (DOI) so that it can be cited independently in the future. For instructions see: <http://journals.plos.org/plosone/s/submission-guidelines#loc-laboratory-protocols>. Additionally, PLOS ONE offers an option for publishing peer-reviewed Lab Protocol articles, which describe protocols hosted on protocols.io. Read more information on sharing protocols at <https://plos.org/protocols?utm_medium=editorial-email&utm_source=authorletters&utm_campaign=protocols>.

We look forward to receiving your revised manuscript.

Kind regards,

Abdelazeem Mohamed Algammal, Prof, Ph.D
Academic Editor
PLOS ONE

Journal Requirements:

1. Please ensure that your manuscript meets PLOS ONE's style requirements, including those for file naming. The PLOS ONE style templates can be found at

https://journals.plos.org/plosone/s/file?id=wjVg/PLOSOne_formatting_sample_main_body.pdf and

<https://journals.plos.org/plosone/s/file?id=ba62/PLOSOne_formatting_sample_title_authors_affiliations.pdf>

 -The entire manuscript was re-checked and re-organized according to the Journal's format.

2. PLOS requires an ORCID iD for the corresponding author in Editorial Manager on papers submitted after December 6th, 2016. Please ensure that you have an ORCID iD and that it is validated in Editorial Manager. To do this, go to ‘Update my Information’ (in the upper left-hand corner of the main menu), and click on the Fetch/Validate link next to the ORCID field. This will take you to the ORCID site and allow you to create a new iD or authenticate a pre-existing iD in Editorial Manager. Please see the following video for instructions on linking an ORCID iD to your Editorial Manager account: <https://www.youtube.com/watch?v=_xcclfuvtxQ>

 -The ORCID ID of corresponding author as (0000-0002-7852-6868) was updated in Editorial manager.

3. Thank you for stating the following in the Acknowledgments Section of your manuscript:

[The authors thank the Department of Microbiology, School of Medicine, Ahvaz Jundishapur University of Medical Sciences, Ahvaz, Iran and Infectious and Tropical Diseases Research Center, Health Research Institute, Ahvaz Jundishapur University of Medical Sciences, Ahvaz, Iran, for financial support (IR.AJUMS.REC.1398.538).]

-The Acknowledgement section was added at the appropriate place in the manuscript as:

"This work is part of a research project which was approved in Infectious and Tropical Diseases Research Center, Ahvaz Jundishapur University of Medical Sciences, Ahvaz, Iran. We are grateful to research affairs of the university for their encouragement and support." **(Page 18)**

We note that you have provided funding information that is not currently declared in your Funding Statement. However, funding information should not appear in the Acknowledgments section or other areas of your manuscript. We will only publish funding information present in the Funding Statement section of the online submission form.

-The funding section was deleted. We will add the grant details in the time of submission of revisions in appropriate place in Editorial Manager.

Please remove any funding-related text from the manuscript and let us know how you would like to update your Funding Statement. Currently, your Funding Statement reads as follows:

 [YES - Specify the role(s) played.]

-The funding details is already removed from manuscript file and will included in Editorial manager in the time of online submission of revisions.

Please include your amended statements within your cover letter; we will change the online submission form on your behalf.

-We included the funding statement in cover letter as well for your information.

4. Please amend either the title on the online submission form (via Edit Submission) or the title in the manuscript so that they are identical.

-We applied the revised title identically in both manuscript file and online submission.

5. Your ethics statement should only appear in the Methods section of your manuscript. If your ethics statement is written in any section besides the Methods, please move it to the Methods section and delete it from any other section. Please ensure that your ethics statement is included in your manuscript, as the ethics statement entered into the online submission form will not be published alongside your manuscript.

-Ethics statement was placed in the beginning of methods section only as below:

"The initial proposal of the work was approved by the Institutional Review Board (IRB) and Ethics Committee of the Ahvaz Jundishapur University of Medical Sciences, Iran, and necessary permission was granted for the work (IR.AJUMS.REC.1398.538)."(**Page 4, lines 84-87)**

6. Please ensure that you refer to Figure 5 in your text as, if accepted, production will need this reference to link the reader to the figure.

- According to revision changes, Figure 5 was removed and the related information was added to Results section in the form of Table 3. **(Page 11)**

7. We note that Figure 5 in your submission contain map images which may be copyrighted. All PLOS content is published under the Creative Commons Attribution License (CC BY 4.0), which means that the manuscript, images, and Supporting Information files will be freely available online, and any third party is permitted to access, download, copy, distribute, and use these materials in any way, even commercially, with proper attribution. For these reasons, we cannot publish previously copyrighted maps or satellite images created using proprietary data, such as Google software (Google Maps, Street View, and Earth). For more information, see our copyright guidelines: http://journals.plos.org/plosone/s/licenses-and-copyright.We require you to either (1) present written permission from the copyright holder to publish these figures specifically under the CC BY 4.0 license, or (2) remove the figures from your submission:

- According to revision changes, Figure 5 was removed

1.    You may seek permission from the original copyright holder of Figure 5 to publish the content specifically under the CC BY 4.0 license.

We recommend that you contact the original copyright holder with the Content Permission Form (http://journals.plos.org/plosone/s/file?id=7c09/content-permission-form.pdf) and the following text:

“I request permission for the open-access journal PLOS ONE to publish XXX under the Creative Commons Attribution License (CCAL) CC BY 4.0 (http://creativecommons.org/licenses/by/4.0/). Please be aware that this license allows unrestricted use and distribution, even commercially, by third parties. Please reply and provide explicit written permission to publish XXX under a CC BY license and complete the attached form.”

Please upload the completed Content Permission Form or other proof of granted permissions as an "Other" file with your submission.

In the figure caption of the copyrighted figure, please include the following text: “Reprinted from [ref] under a CC BY license, with permission from [name of publisher], original copyright [original copyright year].”

2.    If you are unable to obtain permission from the original copyright holder to publish these figures under the CC BY 4.0 license or if the copyright holder’s requirements are incompatible with the CC BY 4.0 license, please either i) remove the figure or ii) supply a replacement figure that complies with the CC BY 4.0 license. Please check copyright information on all replacement figures and update the figure caption with source information. If applicable, please specify in the figure caption text when a figure is similar but not identical to the original image and is therefore for illustrative purposes only.

The following resources for replacing copyrighted map figures may be helpful:

USGS National Map Viewer (public domain): http://viewer.nationalmap.gov/viewer/

The Gateway to Astronaut Photography of Earth (public domain): http://eol.jsc.nasa.gov/sseop/clickmap/

Maps at the CIA (public domain): https://www.cia.gov/library/publications/the-world-factbook/index.html and https://www.cia.gov/library/publications/cia-maps-publications/index.html

NASA Earth Observatory (public domain): http://earthobservatory.nasa.gov/

Landsat: http://landsat.visibleearth.nasa.gov/

USGS EROS (Earth Resources Observatory and Science (EROS) Center) (public domain): http://eros.usgs.gov/#

Natural Earth (public domain): http://www.naturalearthdata.com/

 Table 5 is the work of one of the authors. It has not been removed from the site with a specific source, but it is Photoshop and painting. We assure you that this table has no problem printing

8. Please include a copy of Table 4 and 5 which you refer to in your text on page 7.

-Tables 4 & 5 were re-organized and are now included in the correct place within the revised manuscript. (**Page 12)**

9. Please include captions for your Supporting Information files at the end of your manuscript, and update any in-text citations to match accordingly. Please see our Supporting Information guidelines for more information: <http://journals.plos.org/plosone/s/supporting-information>.

-Our manuscript does not have any Supporting Information files

10. Thank you for submitting the above manuscript to PLOS ONE. During our internal evaluation of the manuscript, we found significant text overlap between your submission and the following previously published works, some of which you are an author.

- https://storage.googleapis.com/plos-corpus-prod/10.1371/journal.pntd.0002573/1/pntd.0002573.pdf?X-Goog-Algorithm=GOOG4-RSA-SHA256&X-Goog-Credential=wombat-sa%40plos-prod.iam.gserviceaccount.com%2F20210514%2Fauto%2Fstorage%2Fgoog4_request&X-Goog-Date=20210514T163734Z&X-Goog-Expires=3600&X-Goog-SignedHeaders=host&X-Goog-Signature=7a819e3dc520f57398bd690bbc02cc3d63414e931d6ed6ce1e2588d4acc0e9d2127ffa246c7b464cfe07870112717cce290e717b1bbd2738efa660f9b9a9a5356f8730a45715763867a438188781c76e2ea84e689d766defdba1e80870ddbfae8449db9aef235621ec8d4dec780dafc95e3808059ef1c9ae87ab61d3838db858f93e9108adcd39825ef2a32be5fea14ac05c01f5e5b58ea27a39336b174bd1fd82ffc3cd53ee3a5e6dc1f8254a9d8e663b73334ccdab3bf743d655ddaf74b9ad007ca1f88a70cc6c1d3d9bc7b76fbbdaacd92aefff061be2a60fb872b6d20e5fdf10198a2a4521647ab6ab59962506f0e6cd037a3b35067a735a8467492694b6

- https://www.ajicjournal.org/article/S0196-6553(14)00995-X/fulltext

- https://www.journalofinfection.com/article/S0163-4453(16)30244-4/fulltext

We would like to make you aware that copying extracts from previous publications, especially outside the methods section, word-for-word is unacceptable. In addition, the reproduction of text from published reports has implications for the copyright that may apply to the publications.

Please revise the manuscript to rephrase the duplicated text, cite your sources, and provide details as to how the current manuscript advances on previous work. Please note that further consideration is dependent on the submission of a manuscript that addresses these concerns about the overlap in text with published work.

We will carefully review your manuscript upon resubmission, so please ensure that your revision is thorough.

-Thank you very much for the opportunity have given us to rewrite the manuscript. Rest assured that we re-checked the entire manuscript phrase-by-phrase.

[Note: HTML markup is below. Please do not edit.]

Reviewers' comments:

Reviewer's Responses to Questions

**Comments to the Author**

1. Is the manuscript technically sound, and do the data support the conclusions?

The manuscript must describe a technically sound piece of scientific research with data that supports the conclusions. Experiments must have been conducted rigorously, with appropriate controls, replication, and sample sizes. The conclusions must be drawn appropriately based on the data presented.

Reviewer #1: Partly

Reviewer #2: Partly

2. Has the statistical analysis been performed appropriately and rigorously?

Reviewer #1: I Don't Know

Reviewer #2: Yes

3. Have the authors made all data underlying the findings in their manuscript fully available?

The [PLOS Data policy](http://www.plosone.org/static/policies.action#sharing) requires authors to make all data underlying the findings described in their manuscript fully available without restriction, with rare exception (please refer to the Data Availability Statement in the manuscript PDF file). The data should be provided as part of the manuscript or its supporting information, or deposited to a public repository. For example, in addition to summary statistics, the data points behind means, medians and variance measures should be available. If there are restrictions on publicly sharing data—e.g. participant privacy or use of data from a third party—those must be specified.

Reviewer #1: Yes

Reviewer #2: No

4. Is the manuscript presented in an intelligible fashion and written in standard English?

PLOS ONE does not copyedit accepted manuscripts, so the language in submitted articles must be clear, correct, and unambiguous. Any typographical or grammatical errors should be corrected at revision, so please note any specific errors here.

Reviewer #1: Yes

Reviewer #2: Yes

5. Review Comments to the Author

Please use the space provided to explain your answers to the questions above. You may also include additional comments for the author, including concerns about dual publication, research ethics, or publication ethics. (Please upload your review as an attachment if it exceeds 20,000 characters)

Reviewer #1: The article title and conclusion do not correlate with the findings in the work done. Furthermore, there are gross grammatical errors and wrong species names in the article. Perhaps another journal would be more suitable for this piece of work.

-The title has been changed as below:

"[**Genotypic and Phenotypic prevalence of *Nocardia* species in Iran: First systematic Review and Meta-Analysis of data accumulated over years 1992-202**](https://www.frontiersin.org/articles/10.3389/fpls.2016.00364/full)**1"**

-The conclusion section is also changed according to the findings

Reviewer #2: The authors determine the prevalence of human nocardia spp. in Iran by using a systematic review and meta-analysis according to the preferred reporting items for systematic reviews and meta-Analyses statement.
It is greatly suggested that the manuscript is accepted after minor revisions. In spite of the scientific value and the medical importance of the investigated pathogen, it lacks the presentation of the overall aspects of this pathogen. I have some comments listed below.
-The title is not representative to the data in the review article and lacks the word of after diversity.
-The review is too short and it is not comprehensive to all ideas relating to the studied subject.

-The title has been changed representing the goal of current work. Besides we were able to extend some topics of the work to make it more comprehensive.

The authors did not detail the phenotypic methods for identification of the investigated pathogen.

-Details of phenotypic methods and biochemical identification tests were added to methods section. **(Page 5-6, line 114-123).** Regarding molecular methods performed and mentioned in articles after 2000, we have also discussed in Discussion section. **(Page 16, lines 307-312)**

-The authors did not talk about the diversity of the pathogen.

-The word "diversity" was removed from the title and the word "prevalence" was added instead. So, we have included articles related to prevalence in all three sections of the results.

" Genotypic and Phenotypic prevalence of *Nocardia* species in Iran:…."

-The authors should detail the pathological importance of the pathogen.

-The pathological importance was added to the text. In Discussion part and a few references was added accordingly **(Page 14 line 263-280)**
-The authors should show the ways to control such pathogen.

-The ways to control Nocardia infections were added to the text in Discussion section **(Page 16, lines 308-314)**

-The authors should detail the diagnostic techniques for the pathogen diseases starting from old to recent. The authors must detail the advantages of each detection methods than others.

-The statement with details were added to discussion section as below:

" In cited surveys conducted before years 2000, the identification system was on the basis of biochemical tests and culture, while after this year, PCR was the method that could differentiate recently identified *Nocardia* spp. Since the first step in controlling of the spread of *Nocardia* spp. and the related infections, is precise detection of species by employing sophisticated laboratory methods, so evidence has reflected that the identification of almost all *Nocardia* isolates were carried out by molecular analysis based on the interconnected *gyr*B-16S rRNA gene sequences; thus, PCR remains the gold standard in this regard (Table 6). " **(Pages 15 & 16, lines 306-312)**

-The authors should talk about the pathogenesis of the microorganisms implicated iwith referring to their virulence factors.

-There were some errors in the structure of several sentences.

-The manuscript has been re-checked in terms of grammar and writing errors.

-The authors must write what each abbreviated word stands for before using the abbreviation for the first time.
-All the family, genus, or species names must be typed italic and its first letter must be capitalized in all the manuscript, but when the microorganism is typed without genus or species, it must be as any normal word without capitalization of its first letter or being italicized.

-Thank you for your help and we made corrections

6. PLOS authors have the option to publish the peer review history of their article ([what does this mean?](https://journals.plos.org/plosone/s/editorial-and-peer-review-process#loc-peer-review-history)). If published, this will include your full peer review and any attached files.


If you choose “no”, your identity will remain anonymous but your review may still be made public.


**Do you want your identity to be public for this peer review?** For information about this choice, including consent withdrawal, please see our [Privacy Policy](https://www.plos.org/privacy-policy).

Reviewer #1: No

Reviewer #2: No

[NOTE: If reviewer comments were submitted as an attachment file, they will be attached to this email and accessible via the submission site. Please log into your account, locate the manuscript record, and check for the action link "View Attachments". If this link does not appear, there are no attachment files.]

While revising your submission, please upload your figure files to the Preflight Analysis and Conversion Engine (PACE) digital diagnostic tool, <https://pacev2.apexcovantage.com/>. PACE helps ensure that figures meet PLOS requirements. To use PACE, you must first register as a user. Registration is free. Then, login and navigate to the UPLOAD tab, where you will find detailed instructions on how to use the tool. If you encounter any issues or have any questions when using PACE, please email PLOS at [figures@plos.org](mailto:figures@plos.org). Please note that Supporting Information files do not need this step.

Dear Dr. khosravi,

Thank you for your PLOS Publication Fee Assistance application for  ().  We acknowledge receipt of your application. As part of our case review process, we will be assessing your application based on the information you provided, your manuscript funding disclosure and your co-author affiliations.  We may contact you to request further information to help us determine your eligibility for Publication Fee Assistance.

Information about an applicant’s application for fee assistance will not be disclosed to journal editors nor reviewers. PLOS publication decisions are based solely on editorial criteria.

We hope to be able to inform you of the disposition of your PFA request within 10 business days of your application.

Kind regards,

PFA Team
